# Differential processing of natural scenes in posterior cortical atrophy and in Alzheimer’s disease, as measured with a saccade choice task

**DOI:** 10.3389/fnint.2014.00060

**Published:** 2014-07-25

**Authors:** Muriel Boucart, Gauthier Calais, Quentin Lenoble, Christine Moroni, Florence Pasquier

**Affiliations:** ^1^Laboratoire Neurosciences Fonctionnelles et Pathologies, Université Lille Nord de France, CNRSLille, France; ^2^Faculté Libre de Médecine, Université Lille Nord de France, Université Catholique de Lille, Service de Neurologie du Groupement des Hôpitaux de l’Institut Catholique de LilleLille, France; ^3^Centre de la Mémoire, Centre Hospitalier Universitaire de Lille, Université Lille Nord de FranceLille, France

**Keywords:** Alzheimer, posterior cortical atrophy, context, scene perception, saccades

## Abstract

Atrophy of the medial temporal lobe structures that support scene perception and the binding of an object to its context (i.e., the hippocampus and the parahippocampal cortex) appears early in the course of Alzheimer’s disease (AD). However, few studies have investigated scene perception in people with AD. Here, we assessed the ability to find a target object within a natural scene in people with AD and in people with posterior cortical atrophy (PCA, a variant of AD). Pairs of color photographs were displayed on the left and right of a fixation cross for 1 s. In separate blocks of trials, participants were asked to categorize the target (an animal) by either moving their eyes toward the photograph containing the target (the saccadic choice task) or pressing a key corresponding to the target’s location (the manual choice task). Isolated objects and objects within scenes were studied in both tasks. Participants with PCA were more impaired in detection of a target within a scene than participants with AD. The latter’s performance pattern was more similar to that of age-matched controls in terms of accuracy, saccade latencies and the benefit gained from contextual information. Participants with PCA benefited less from contextual information in both the saccade and the manual choice tasks—suggesting that people with posterior brain lesions have impairments in figure/ground segregation and are more sensitive to object crowding.

Although memory deficits are typically the first symptoms of Alzheimer’s disease (AD) to appear, a growing body of evidence suggests that many aspects of visual cognition are impaired in people with AD (Mendola et al., 1995; Valenti, 2010). Indeed, some studies indicate that visual disturbances might even precede the memory deficits (Benson et al., 1988) and may be predictive of cognitive impairments (Cronin-Golomb et al., 1995). Furthermore, impaired object perception affects instrumental activities of daily living in people with AD (Mosimann et al., 2004; Jefferson et al., 2006). Disturbances in both spatial and object recognition processes have been reported consistent with the impact of the disease on both dorsal stream areas, in posterior cortical atrophy (PCA), and atrophy of ventral stream areas (Possin, 2010) though impairments in functions of the ventral stream seem more severe in AD. In contrast, ventral stream aspects of visual cognition, such as recognizing objects and faces, tend to be less impacted than dorsal stream aspects such as mental rotation, coherent motion perception or angle discrimination in PCA than in AD (Possin, 2010). However, the pattern of visual impairments in patients with PCA is heterogeneous (Tsai et al., 2011). PCA has been defined as a “nontypical form of Alzheimer disease” or as a “visual variant” of AD. It is characterized by a relatively selective decline in visual processing and other posterior cortical functions (such as visuomotor and visuospatial abilities), whereas impairments of memory, language and other cognitive functions only occur late in the disease (Benson et al., 1988; Schmidtke et al., 2005; McMonagle et al., 2006; Lehmann et al., 2011). Neuroimaging studies shown that atrophy in PCA is more marked in posterior regions of the brain (the parietal, temporal and occipital cortices) (Tenovuo et al., 2008; Kennedy et al., 2012; Migliaccio et al., 2012), which results in a range of visual disturbances (including visual agnosia, environmental disorientation, apraxia and alexia) (Mendez et al., 2002; Crutch et al., 2012).

Object recognition experiments, whether performed with healthy subjects or patients, typically investigate objects in isolation. However, “real-world” objects rarely appear in the absence of some context. Few studies have investigated scene perception in people with AD. This is paradoxical, given (i) the early atrophy of the medial temporal lobe (Delacourte et al., 1999; Villain et al., 2008), which can start at least 3 years before AD is diagnosed (Ridha et al., 2008); and (ii) significant cell loss (Davies, 2006) in structures (the hippocampus and parahippocampal cortex) that support scene perception (Epstein and Kanwisher, 1998; Epstein, 2008) and the binding of an object to its usual context (Goh et al., 2004; Fenske et al., 2006). Lee et al. (2007) found that patients with AD were more impaired in the discrimination of scenes (either landscapes or in virtual reality rooms), than in the discrimination of faces. Shakespeare et al. (2013) examined scene perception in patients with PCA. In a description task, patients named fewer features and made more misperceptions than controls. When visual exploration was recorded, the researchers observed that a number of patients fixated on uninformative parts of the scene rather than the details that were commonly fixated by controls. This was interpreted as evidencing poor eye movement control and/or an inability to implement a successful scanning strategy.

Here, we used a saccadic choice task to investigate scene perception and, more specifically, the ability to find a target object within a natural scene in a group of people with AD and a group with PCA. The saccadic choice paradigm was initially developed by Kirchner and Thorpe (2006) to measure the speed of object categorization. In their original study, Kirchner and Thorpe presented healthy young adult participants with two lateral (left/right) photographs of natural scenes and asked them to move their eyes as quickly as possible to the scene containing an animal. The researchers found that although the median latency was 228 ms, the fastest saccades to animal targets were triggered as soon as 120–130 ms after stimulus onset. Crouzet et al. (2010) subsequently showed that these rapid saccadic responses can be initiated even more rapidly (around 100 ms post-onset) when the target is a human faces and more slowly when the target is a non-biological object (e.g., a vehicle). Crouzet and Thorpe (2011) then suggested that the underlying mechanism for such rapid responses may be based on information that is only partially processed by the occipitotemporal cortex (Cauchoix and Crouzet, 2013) (possibly in V4) (Crouzet et al., 2012).

In the present study, participants were shown two photographs (presented to the left and right of a central fixation point) and asked to saccade to the image containing an animal. To assess the patients’ ability to process contextual information, we compared their performance under two different conditions: the target animal was presented either in isolation on a homogeneous gray background or in its natural setting. Given that impairments in eye movement (e.g., prolonged saccade latencies and hypometric saccades) have been reported in patients with AD (Fletcher and Sharpe, 1986; Shafiq-Antonacci et al., 2003), the saccade responses were compared with manual responses using the same stimuli and the same presentation conditions.

A growing body of literature evidence from behavioral, visual cognition (Biederman et al., 1982; Boyce et al., 1989; Boyce and Pollatsek, 1992; de Graef et al., 1990; Henderson et al., 1999; Davenport, 2007), electrophysiology (Ganis and Kutas, 2003) and brain-imaging studies (Bar and Aminoff, 2003; Goh et al., 2004; Kirk, 2008; Mudrick et al., 2010) in healthy people suggests that contextual information affects the efficiency of object searching and recognition. We therefore expected the presence of context to facilitate target detection in healthy participants. As the parahippocampal region is involved in the binding of an object to its context (Bar, 2004) and this region is affected early by cellular and structural alterations in AD (Ridha et al., 2008; Villain et al., 2008; Apostolova et al., 2012), we expected patients with AD to benefit less than healthy participants from the background. Indeed, parahippocampal atrophy is even considered as an early biomarker for AD (Echávarri et al., 2011). The impairment of basic visual skills (including visual acuity, line orientation, contour integration, figure/ground segregation, form detection and discrimination, motion discrimination and point localization) in patients with PCA (Metzler-Baddeley et al., 2010; Lehmann et al., 2011) is consistent with evidence of widespread lesions within the occipital and parietal areas in this disease. Hence, we expected patients with PCA to be more sensitive than patients with AD to (i) lateral masking of the object by the background; and (ii) crowding effects (Crutch et al., 2012) and thus more impaired than patients with AD when the target object was embedded within a scene (relative to embedding in a homogeneous background).

## Method

### Participants

Six patients (3 males) with a diagnosis of PCA, 14 patients (6 males) with a diagnosis of AD, 15 healthy elderly adults (5 males) and 10 healthy young adults (3 males) were enrolled in the study by Lille University Hospital’s Memory Clinic (Lille, France). Patients taking cholinesterase inhibitors were included if the dose had not changed for at least 6 weeks prior to inclusion. Despite the absence of ophthalmologic impairments or psychiatric disorders, all the patients with PCA had progressive, insidious signs of impaired visuospatial orientation. Magnetic resonance imaging (MRI) was used to confirm the atrophy of the occipital, parietal and (in some cases) temporal cortex and the absence of hippocampal atrophy. Patients with PCA were diagnosed according to the criteria published by Tang-Wai et al. (2004).

The major initial symptom in patients with AD was a progressive memory complaint (for at least 6 months previously) whose symptoms interfered with activities of daily living. MRI showed predominant hippocampal atrophy. All patients fulfilled the International Working Group’s research criteria (Dubois et al., 2010) after a comprehensive work-up including a neuropsychological assessment, MRI, CSF biomarker assays and SPECT or FDG-PET.

On average, the patients with AD were older than the patients with PCA and the elderly adult controls (mean ± SD age: 71.5 ± 10, 65.4 ± 5 and 66 ± 7, respectively) but the differences in age were not statistically significant. In the PCA group, the mean ± SD Mini Mental State Examination (MMSE; Folstein et al., 1975) and Mattis Dementia Score (DRS; Mattis, 1973) scores were 22.5 ± 3.61 and 114.5 ± 13.63, respectively. In the AD group, the mean MMSE and DRS scores were 23.3 ± 1.34 and 112.42 ± 24.55, respectively. There were no significant inter-group differences in these scores. Patients were excluded if they had evidence of vascular lesions, major depression or ophthalmologic impairments (cataract, macular degeneration or glaucoma). Patients with visual agnosia and/or hemineglect were also excluded. The control group was composed of elderly volunteers recruited from among the patients’ relatives and had a mean ± SD MMSE score of 29.9 ± 0.2. Ophthalmological screening included a detailed review of current or past visual disturbances, the assessment of visual acuity, the Amsler grid (for macular degeneration) and signs of cataract. The young adult controls (mean age: 29.6 ± 8.5) were students in medicine or neuroscience and none had a history of neurological or psychiatric disease. The study was approved by the local investigational review board (CPP Nord-Ouest IV, reference 05/79) and performed in accordance with the tenets of the Declaration of Helsinki. Written, informed consent was provided by all participants.

### Stimuli

**Figure 1:**
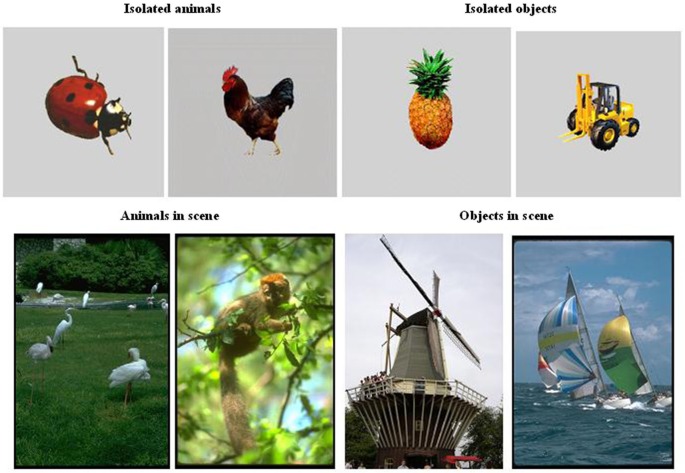
**Examples of stimuli (animals and objects) used in the categorization tasks (saccade and manual responses)**.

The stimuli were photographs of either natural scenes or isolated objects selected from commercially available libraries (Corel and Hemera Photo-Objects, respectively). Examples are shown in Figure 1. At a viewing distance of 57 cm, the mean angular size of the stimuli was 7° vertically × 5° horizontally for photographs of scenes and 5° × 5° for photographs of isolated objects. All pictures were displayed on a black background. Target animals included mammals, reptiles, insects, birds, crustaceans, and fish. The distractors were variously houses, monuments, means of transportation, flowers, fruits, and landscapes containing neither animals nor humans.

### Equipment

Eye movements were recorded by a remote pupil-tracking system (RED-m, Senso-Motoric Instruments, Berlin, Germany) that uses infrared illumination and computer-based image processing. The tracking system records the eye position at a sampling rate of 120 Hz and compensates for slight head movements. The manufacturers report a gaze position accuracy of 0.5°. Images of the eye are analyzed in real-time by detecting the pupil, calculating the center and eliminating artifacts. Movement data were collected for each eye separately. The calibration stimulus was a grid containing nine white dots (2° × 2° degrees) displayed one at a time on a black background. During calibration, the participants were instructed to fixate the dot located in the middle of the screen and to move their eyes to the other dots as instructed. Calibration was performed twice, in order to check the stability. The experimental trials were only initiated if the eye tracker classified the calibration as “good”. The participants were given the same instructions for all experimental trials: to look at the central fixation cross with their gaze as steady as possible. The pictures were displayed with Experiment Center software (Senso-Motoric Instruments). The recorded eye movement data were analyzed with BeGaze software (Senso-Motoric Instruments). Manual responses were recorded via a box fitted with two keys and connected to the computer.

### Procedure

Each participant was tested in two experimental sessions. The categorization tasks were composed of four separate blocks of trials determined by the response (saccade vs. manual) and the type of picture (isolated object vs. object in a scene). There were 100 trials in each block: 50 trials with the animal on the right of the fixation cross and 50 trials with the animal on the left. To reduce priming effects, the saccade and manual response tasks were separated by at least 1 week and the stimuli were randomly selected from among 1000 scenes containing animals, 1000 scenes without animals, 200 isolated animals and 200 isolated objects. The fixation cross was displayed for 500 ms. Then, 200 ms after disappearance of the cross, a pair of photographs were simultaneously presented for 1 s (one located 7° left of the center of fixation and the other located 7° right of the center). The gap period of 200 ms between the disappearance of the fixation cross and the appearance of the photographs usually accelerates saccade initiation (Masson et al., 2000). In two blocks of 100 trials each (one with scenes as stimuli and the one with isolated objects) the participants were asked to saccade to the picture containing an animal. The left-side or right-side position of the target was randomized. Half of the participants in each group started with the isolated objects and the other half started with the scenes. In two other blocks of 100 trials each, the response was manual; the procedure was the same as in the saccade task but participants were instructed to respond by pressing the right key or the left key, depending on the target animal’s location.

## Results

### The saccadic categorization task

**Figure 2:**
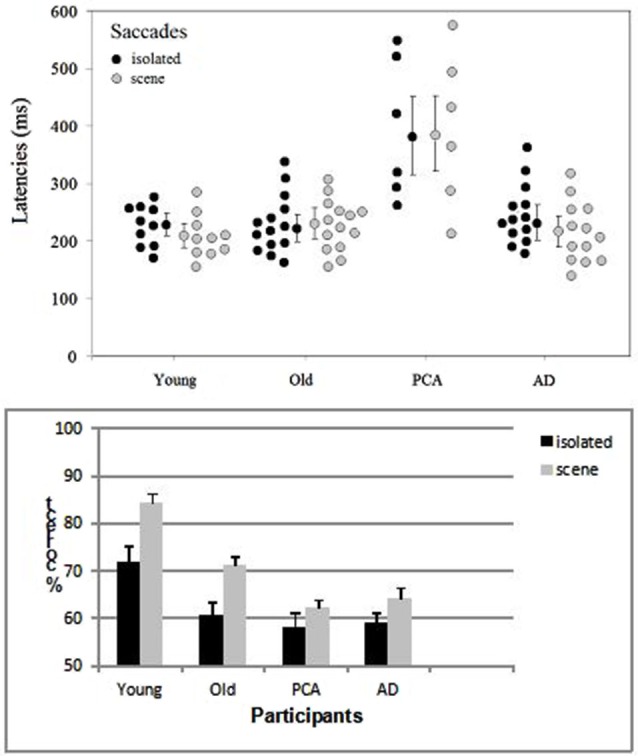
**Distribution of the mean saccade latency (with each group’s median and standard error) and accuracy (with the standard error) as a function of group (young adult controls, elderly adult controls, patients with AD and patients with PCA) and the type of image (isolated targets vs. targets in scenes)**.

Participants whose overall performance differed by two SDs from the mean were discarded. One patient with AD was excluded from the analysis because of performance at ceiling (i.e., a correct response rate of 96%) and one healthy elderly control was excluded because of performance at chance. Saccade latencies below 100 ms were considered to be anticipatory and were excluded from the analysis. Response accuracy and saccade latencies for correct responses were examined in analyses of variance (ANOVAs, performed with Statistica software). The target’s spatial location (left/right), the group (young adult controls, elderly adult controls, people with AD, people with PCA) and the category of image (scenes/isolated objects) were included as variables (Figure 2). The participants in each group were the random variable.

The only difference between left and right targets was seen in the young adult controls (*t*_(9)_ = 2.59, *p* < 0.029), who displayed greater accuracy for targets on the left (87.8%) than on the right (81%) in the scene condition. The effect of group was significant both for saccade latencies (*F*_(3, 39)_ = 11.14, *p* < 0.001) and accuracy (*F*_(3, 39)_ = 12.6, *p* < 0.001). The young adult controls and elderly adult controls did not differ significantly in terms of the saccade latency (respectively 228 and 226 ms in the isolated object condition and 223 and 229 ms in the scene condition). However, the accuracy rate was 12.4% greater in young adult controls than in older participants (*F*_(1, 22)_ = 17.4, *p* < 0.001). Patients with AD did not differ significantly from age-matched controls in terms of either latency (240 vs. 228 ms, respectively; *F*_(1, 25)_ = 0.3, *p* = 0.61) or accuracy (61.6% vs. 65.9%, respectively; (*F*_(1, 25)_ = 2.4, *p* = 0.12), except when scenes were used as stimuli (*F*_(1, 25)_ = 5.97, *p* < 0.05). Patients with PCA were slower (by 153 ms, *F*_(1, 18)_ = 28.2, *p* < 0.001) and less accurate (by 5.6%) than age-matched controls. The difference in accuracy was not significant *F*_(1, 18)_ = 2.6, *p* = 0.11), except when scenes were used as stimuli (*F*_(1, 18)_ = 5.9, *p* < 0.05). Patients with PCA were also slower than patients with AD (by 141 ms; *F*_(1, 17)_ = 23.5, *p* < 0.001) but not significantly less accurate (60.3% and 61.6%, respectively). When averaged over all four groups, saccade latencies were similar for targets in their natural scenes (268 ms) and for isolated targets (269 ms) but accuracy was greater for targets in scenes than for isolated targets (70.6% vs. 62.4%, respectively; *F*_(1, 39)_ = 25.7, *p* < 0.001). This difference was observed (see Figure 2) for all groups but was only statistically significant for controls. The difference was 4.3% for patients with PCA, 5.6% for patients with AD, 10.6% (*t*_(13)_ = 4.1, *p* < 0.001) for elderly controls and 12.4% (*t*_(9)_ = 7, *p* < 0.001) for young adult controls. The group × type of image interaction did not achieve statistical significance.

### The manual categorization task

**Figure 3:**
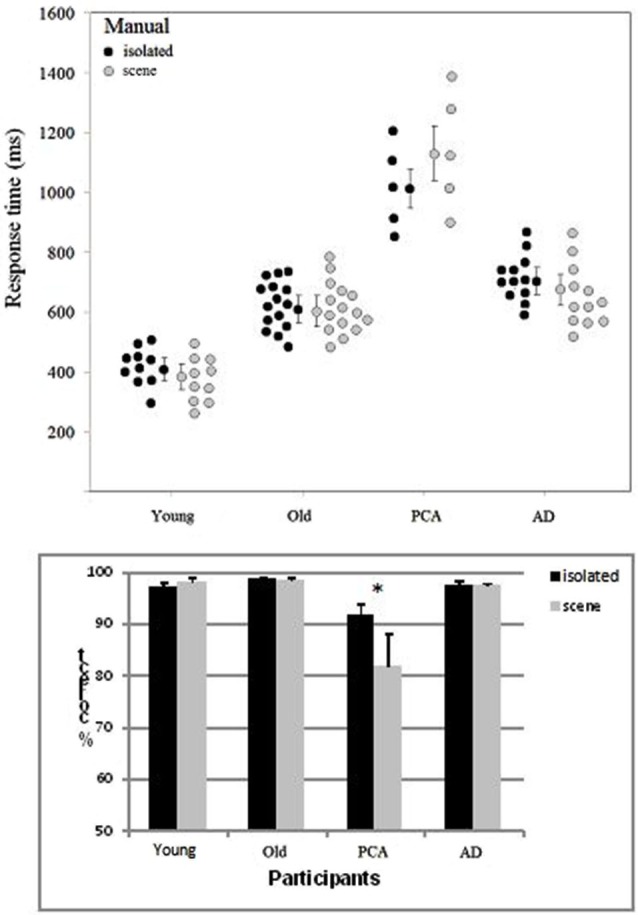
**Distribution of the mean manual RT (with each group’s median and standard error) and accuracy (with the standard error) as a function of group (young adult controls, elderly adult controls, patients with AD and patients with PCA) and the type of image (isolated targets vs. targets in scenes)**.

Participants whose overall performance differed by two SD values or more from the mean were excluded from the analysis. Two patients with AD were excluded because of slow Response times (RTs). One of the participants with PCA failed to attend the session including the manual categorization task. RTs below 100 ms were excluded. Accuracy and correct RTs were examined in ANOVAs. The target’s spatial location (left/right), the group (young adult controls, elderly adult controls, people with AD, people with PCA) and the category of image (scenes/isolated objects) were included as variables (Figure 3). The participants in each group were the random variable.

The target’s location (left/right) did not have a significant main effect on either accuracy or the RT in any of the four groups of participants. The effect of group was significant both for accuracy (*F*_(3, 38)_ = 13.6, *p* < 0.001) and the RT (*F*_(3, 38)_ = 52.6, *p* < 0.001). Participants with PCA were slower than participants with AD (by 447 ms; *F*_(1, 15)_ = 39.2, *p* < 0.001) and less accurate (by 10.6%; *F*_(1, 15)_ = 29.1, *p* < 0.001). They were also less accurate (*F*_(1, 18)_ = 38.1, *p* < 0.001) and slower (*F*_(1, 18)_ = 77.8, *p* < 0.001) than age-matched controls. As can be seen in Figure 3, participants with AD were slower than healthy, elderly, age-matched controls (by 127 ms; *F*_(1, 25)_ = 10.0, *p* < 0.001) but were not less accurate (97.5 and 98.6%, respectively). Young adult controls were faster than elderly controls (by 215 ms; *F*_(1, 23)_ = 25.6, *p* < 0.001) but were not less accurate (97.6% and 98.6%, respectively).

In contrast to the results for the saccade response task, accuracy in the manual categorization task was better for isolated animals (96.3%) than for animals in scenes (94% *F*_(1, 38)_ = 10.7, *p* < 0.002). RTs were shorter for isolated animals than for animals in scenes (by 18 ms, *F*_(1, 38)_ = 1.4, ns). The category of image interacted significantly with group for both the RTs (*F*_(3, 38)_ = 2.93, *p* < 0.04) and accuracy (*F*_(3, 38)_ = 9.58, *p* < 0.001). As can be seen in Figure 3, this was mainly due to participants with PCA, who responded more rapidly and more accurately for isolated targets than for scenes (isolated: 1009 ms and 91.9%; scenes: 1142 ms and 81.9%; accuracy (*F*_(1, 4)_ = 30.1, *p* < 0.001; RTs: *F*_(1, 4)_ = 7.5, *p* < 0.01)). There were no significant differences in accuracy or RTs when the two image categories were compared in the three other groups of participants.

When averaged across all four groups and the two types of image, accuracy was higher in the manual categorization task than in the saccade categorization task (95.2% and 66.5%, respectively; *F*_(1, 37)_ = 442, *p* < 0.001) but manual RTs were longer than saccade latencies (708 vs. 269 ms, respectively; *F*_(1, 37)_ = 635.8, *p* < 0.001).

## Discussion

In the present study, we investigated scene perception (and, more specifically, the use of background contextual information on object categorization) in people with AD and people with PCA (a variant of AD affecting the occipitoparietal regions of the brain).

In view of the early-onset lesions in the hippocampal area in AD and in the occipitoparietal areas in PCA, we expected both populations to be impaired when compared with age-matched controls. Indeed, our analysis of the saccade response task (during which participants had to respond quickly) showed that both patients with AD and patients with PCA were less accurate than age-matched controls when scenes were used as stimuli. In the manual response task, participants had more time to explore the images. If the first saccade went to the wrong side (i.e., to the image lacking an animal), the participant was able to shift his/her gaze to the other side before giving a manual response. Under this condition, patients with AD no longer displayed an impairment in accuracy (the correct response rate was over 95%) but were still slower than age-matched controls. Patients with PCA were always less accurate than the other groups—especially when scenes were used as stimuli. When given more time to respond (i.e., in the manual response task), the patients with PCA had markedly better performance levels when isolated objects were used as stimuli.

The lower observed accuracy in the AD group (relative to age-matched controls) when scenes were used as stimuli in the saccade choice task confirms the results of previous studies (Boucart et al., 2014) in which scenes were the only stimuli used. Our results for the saccade choice task show that the three groups of older participants were particularly impaired (with a correct response rate below 60%) when isolated objects served as stimuli. Performance was better for scenes in all groups. This suggests that in a rapid choice task, the background context facilitates selection of the target. When participants are given more time to explore images (as in the manual response task), the background context has less effect: performance at ceiling was observed in all groups except for the PCA group. Worse performance for isolated objects than for scenes in the saccade task cannot be explained by the size of the target object because the animals in scenes were no larger than animals displayed in isolation. It cannot be held that isolated animals were more difficult to categorize than animals in scenes because performances for the two types of image were similar in the manual response task (except in the PCA group). It is likely that the context facilitated performance when there was limited time for providing a response. The results of studies of contextual information processing in healthy young adults suggest that background information is processed early (possibly by the fast magnocellular pathway). For instance, Bar (2004) suggested that (i) if background (contextual) information is to assist the recognition process, it has to be extracted rapidly; and (ii) this rapid extraction is mediated by general cues conveyed by low spatial frequencies in the image. This coarse information is projected rapidly from the visual cortex to the prefrontal and parahippocampal cortices (possibly via the magnocellular pathway), where it can activate a scene schema. The representation is then refined and further instantiated as specific details progressively arrive at higher spatial frequencies. Magnocellular dysfunction (as demonstrated by electroretinography and visual-evoked potentials for simple stimuli (gratings varying in spatial and temporal frequencies) has been reported in AD (Gilmore and Whitehouse, 1995; Jacobs et al., 2002; Sartucci et al., 2010). Lenoble et al. (2013) found that impairments in the magnocellular pathway affect high-level object categorization.

As can be seen in Figures 2, 3, the differences between the AD group and age-matched controls in both the saccade and manual categorization tasks were smaller than the differences between the PCA group and age-matched controls. Saccade latencies and manual RTs were longer in patients with AD than in age-matched controls; this agrees with reports of greater overall processing times in AD (Shafiq-Antonacci et al., 2003). Analyses of dependent variables (accuracy and response time) in elderly controls often reveal a speed-accuracy trade-off. This has been interpreted as the use of a more cautious response strategy in order to avoid errors or to compensate for decreased cognitive control (Ratcliff et al., 2007; Endrass et al., 2012). In the present study, the AD patients’ results may be due to a compensatory shift in response strategy. Nevertheless, the AD group’s excellent accuracy (>90%) in discriminating a target animal presented for a limited presentation time (1 s) in the manual response task contrasts with the results of Neargarder and Cronin-Golomb (2005) study. They showed that patients with AD were impaired at detecting a change within pairs of scenes. However, Neargarder and Cronin-Golomb’s patients with AD were older than those in the present study (with mean ages of 80.4 and 71, respectively) and had lower MMSE scores (19.5 and 23, respectively). Moreover, the detection of a change requires more attention to local parts of the scene than the detection of an animal within a scene; the latter task can be accomplished with low-resolution peripheral vision (at an eccentricity of 75°) (Thorpe et al., 2001). Scinto et al. (1994) showed that patients with AD are more impaired in tasks that place increased demands on attention.

Our patients with PCA were slower and less accurate than age-matched controls in the saccade task (especially when scenes served as the stimuli) but, in contrast to patients with AD, they were also less accurate and slower than both age-matched controls and participants with AD in the manual response task. Another major difference between patients with PCA on one hand and patients with AD and controls on the other is that the former exhibited better performance levels for isolated animals than for animals in scenes in the manual response task. Shakespeare et al. (2013) presented objects, faces and scenes in separate blocks of trials to patients with PCA and to age-matched controls. As in the present study, Shakespeare et al. observed a better performance for objects than for scenes in patients with PCA. However, in contrast to our present study, controls also had better performance levels for objects than for scenes. When considering Shakespeare et al.’s study in more detail, it is noteworthy that participants were shown a single image on each trial and asked to choose the corresponding name in a three-alternative, forced verbal categorization task (e.g., “Is this a forest, a desert or a beach?”). Participants may be more likely to confuse a desert and a beach than they are to fail to detect an animal in a pair of scenes. The low impact of contextual information in patients with PCA (relative to the other groups of participants) might reflect a higher sensitivity to crowding, and impaired figure/ground segregation, in this population. Consistently, Shakespeare et al. (2013) reported that patients with PCA responded more accurately and more rapidly to colored stimuli than they did to gray-scale. Color is known to facilitate the segmentation of surfaces and figure/ground segregation—especially when vision is impaired experimentally (Oliva and Schyns, 2000) or by disease (Wurm et al., 1993; Boucart et al., 2008). Crowding refers to the decreased visibility of a visual target in the presence of nearby objects or structures; it impairs the ability to recognize objects in “cluttered” scenes and has a more pronounced effect on peripheral vision (Pelli et al., 2004; Levi, 2008). Consistently, Crutch and Warrington (2007) reported high sensitivity to crowding in two patients with PCA in a letter reading task in which the spatial proximity of a flanker was manipulated. In the people with PCA assessed in our present study, both crowding and difficulties in figure/ground segregation were more problematic for scenes than for isolated objects. In monkeys, figure/ground segregation is reportedly altered by lesions to the visual cortex (Supèr and Lamme, 2007).

With the exception of accuracy, there were no differences in oculomotor parameters when comparing young and elderly adult controls. Although many studies have observed effects of aging on eye movements (Irving et al., 2006; Porter et al., 2010; Paquette and Fung, 2011; Ridderinkhof and Wijnen, 2011), it seems that automatic parameters (such as latencies in pro-saccade tasks) are barely influenced by aging (Abrams et al., 1998; Munoz et al., 1998; Kaneko et al., 2004).

Conclusion: Literature studies of object and scene perception in AD have not usually distinguished between typical AD and atypical AD (i.e., forms with lesions in the posterior parts of the brain, such as PCA). Our present results revealed differences in performance in patients with PCA, who were more impaired in scene perception than patients with AD. The latter displayed performance patterns (in terms of accuracy and the benefit gained from contextual information) that were more similar to those in age-matched controls. Patients with PCA benefit less from contextual information, suggesting higher sensitivity to crowding and impaired figure/ground segregation in people with lesions in posterior areas of the brain.

## Conflict of interest statement

The authors declare that the research was conducted in the absence of any commercial or financial relationships that could be construed as a potential conflict of interest.
